# Glucose Augments Killing Efficiency of Daptomycin Challenged *Staphylococcus aureus* Persisters

**DOI:** 10.1371/journal.pone.0150907

**Published:** 2016-03-09

**Authors:** Marcel Prax, Lukas Mechler, Christopher Weidenmaier, Ralph Bertram

**Affiliations:** 1 Interfakultäres Institut für Mikrobiologie und Infektionsmedizin, Lehrbereich Mikrobielle Genetik, Auf der Morgenstelle 28, Eberhard Karls Universität Tübingen, 72076 Tübingen, Germany; 2 Paul-Ehrlich-Institut, Mikrobiologische Sicherheit, Paul-Ehrlich-Str. 51–59, 63225 Langen, Germany; 3 Interfakultäres Institut für Mikrobiologie und Infektionsmedizin, Medizinische Mikrobiologie und Hygiene, Elfriede-Aulhorn-Str. 6, Eberhard Karls Universität Tübingen, 72076 Tübingen, Germany; 4 Klinikum Nürnberg Medical School GmbH, Research Department, Paracelsus Medical University, Nuremberg, Germany; University of Groningen, Groningen Institute for Biomolecular Sciences and Biotechnology, NETHERLANDS

## Abstract

Treatment of *Staphylococcus aureus* in stationary growth phase with high doses of the antibiotic daptomycin (DAP) eradicates the vast majority of the culture and leaves persister cells behind. Despite resting in a drug-tolerant and dormant state, persister cells exhibit metabolic activity which might be exploited for their elimination. We here report that the addition of glucose to *S*. *aureus* persisters treated with DAP increased killing by up to five-fold within one hour. This glucose-DAP effect also occurred with strains less sensitive to the drug. The underlying mechanism is independent of the proton motive force and was not observed with non-metabolizable 2-deoxy-glucose. Our results are consistent with two hypotheses on the glucose-DAP interplay. The first is based upon glucose-induced carbohydrate transport proteins that may influence DAP and the second suggests that glucose may trigger the release or activity of cell-lytic proteins to augment DAP’s mode of action.

## Introduction

Eradication of harmful bacteria in the human body is often cumbersome due to drug resistance and drug tolerance particularly in biofilm embedded cells [1–7]. Biofilms accommodate a high percentage of persister cells which are in a non-dividing and metabolically less active state [8]. Persisters are regarded as genetically identical variants among a population of unicellular organisms that tolerate and survive high concentrations of antibiotics over extended periods of time [9–12]. This kind of phenotypic heterogeneity is a successful bet-hedging strategy to endure hostile conditions, such as antibiotic treatment or immune response and provides a rationale for recurrent or chronic bacterial infections [9,13,14]. The level of persister cells among a clonal bacterial culture is influenced by nutrient limitation, growth phase, various stresses, quorum sensing and other factors [15–17]. Compared to the identification of numerous persister-genes, information available on metabolic aspects of persisters is more limited [18]. A change in carbon source utilization upon glucose limitation stimulates persister formation in *E*. *coli* [19] and accordingly, *E*. *coli* persisters maintain glycerol and glucose metabolism [20–22]. *De novo* synthesis of amino acids was observed with persister cells of the notorious pathogen *Staphylococcus aureus* [23], which is causative of skin infections, osteomyelitis, endocarditis, bacteremia and further illnesses [24–27]. Multiple antibiotic resistant *S*. *aureus* strains continue to pose a formidable challenge in hospitals and in the community [28]. The bactericidal lipopeptide daptomycin (DAP) is one of few antibiotics that is generally effective against many *S*. *aureus* strains [29], as well as other Gram positive bacteria [30–32]. The amphiphilic character of DAP in combination with calcium cations facilitates the incorporation into the bacterial membrane [33]. According to the current model, oligomerization of DAP leads to pore formation and increased permeability for ions resulting in perturbation of the proton motive force (PMF) and cell death [34]. DAP is highly efficient also against *S*. *aureus* cells in stationary phase, which are tolerant towards a broad range of other antibiotics [35]. As shown previously, the eradication efficiency of *S*. *aureus* by DAP is enhanced upon combination with other antibiotics [36,37] or D-cycloserine [38]. First cases of DAP non-susceptible strains were documented in hospitals briefly after introduction of the drug [39]. Such strains frequently exhibit changes in the cell envelope [40–42]. To prevent resistance formation and selection for non-susceptible strains due to prolonged drug-treatments [7], it is necessary to develop new efficient therapeutic strategies, with a special focus on targeting persister cells [43].

A new means for persister eradication in biofilms was achieved by a combination therapy with rifampicin and the acyldepsipeptide antibiotic ADEP4, leading to the permanent activation of protease ClpP [44]. Furthermore, the administration of carbohydrates increases persister killing by aminoglycosides due to their dependency on the proton motive force (PMF) [45,46]. We here show that supplementing cultures of DAP challenged *S*. *aureus* cells with specific carbohydrates *in vitro* leads to accelerated killing, which intriguingly also pertains to strains less susceptible to this drug. According to our data, the underlying mechanism is not-dependent on the PMF but may be dependent on metabolization of glucose. Unraveling the molecular basis and exploiting this phenomenon provides perspectives for a powerful anti-persister therapy.

## Materials and Methods

### Bacteria, growth conditions, and working solutions

Bacterial strains used in this study are listed in Table 1. Unless stated otherwise, cultures were incubated at 37°C with aeration and shaking (150 rpm) in tryptic soy broth (TSB). To provide stationary growth phase cultures, incubations were performed overnight. In the course of this study, two different types of TSB were used, either composed of casein peptone (pancreatic) (17 g/L), soy peptone (A3SC) (3 g/L), NaCl (5 g/L), K_2_HPO_4_ (2.5 g/L) and glucose (2.5 g/L, sterile filtered and added after autoclaving, or a ready-to-use powder purchased from Sigma, both at an approximate 1:10 culture-to-flask volume ratio. Daptomycin (DAP, analytic grade powder; designated ‘Cubicin’, Novartis Pharma, Nuremberg, Germany) was prepared freshly prior to each application, filter sterilized (0.2 μM pore size, Whatman, Dassel, Germany) and used to challenge stationary-phase *S*. *aureus* cells. 100-fold the MIC of DAP solution had been determined as 400 mg/L for *S*. *aureus* SA113 before [35] and 150 mg/L corresponding to 250-fold the MIC was determined and used for NARSA strains [47]. Strain HG003 D6 [48] was treated with 400 mg/L corresponding to 100-fold the MIC and the clinical strains with 400 mg/L corresponding to 200-fold and 800-fold the MIC for strains 616/621 and 701/703 respectively. Ca^2+^ cations, required for DAP activity [49], were provided as CaCl_2_ (Merck, Darmstadt, Germany) at a final concentration of 50 μg/mL.

**Table 1:** List of strains used in this study.

*S*. *aureus* strains	Description	Reference
SA113 (ATCC 35556)	NCTC8325 derivative, *agr*^-^,*rsbU*^*-*^. *agr*: accessory gene regulator quorum-sensing system; *rsbU*: positive regulator of σ^B^	[50]
MSSA 616, 621	clinical isolates, DAP MIC, 0.5 mg/L	[42]
MSSA 701, 703	clinical isolates, DAP MIC, 2.0 mg/L	[42]
HG003	NCTC8325 derivative, *rsbU* and *tcaR* repaired	[51]
HG003 D6	Derivative of HG003 carrying SNP in SAOUHSC_00670 (*pitA*) and SAOUHSC_02622 (*gltS*).	[48]
USA300 NE39	*ptsG*^*-*^ (phosphotransferase system, glucose-specific IIABC component)	[47]
USA300 NE172	*ptsG*^*-*^ (phosphotransferase system, glucose-specific IIABC component)	[47]
USA300 NE427	*fumC*^*-*^ (fumarate hydratase)	[47]
USA300 NE476	*fbaA*^*-*^ (fructose bisphosphate aldolase)	[47]
USA300 NE491	*icd*^*-*^ (isocitrate dehydrogenase)	[47]
USA300NE1003	*mqo*^*-*^ (malate:quinone oxidoreductase)	[47]
USA300 NE1046	*fdh*^*-*^ **(**formate dehydrogenase accessory protein)	[47]
USA300 NE1407	*pyk*^*-*^ (pyruvate kinase)	[47]

### MIC determination

The MIC of all strains used in this study towards DAP was determined as described before [35] in triplicates with at least two independent cultures grown in TSB medium. The MIC was defined as the lowest antibiotic concentration that inhibited growth after 24 hours incubation at 37°C without shaking.

### Glucose and acetate determination

Glucose and acetate concentrations in culture supernatants were both determined using enzymatic kit systems (R-Biopharm, Art. No. 10716251035, Art. No. 10148261035) according to the manufacturer’s instructions but with the following modifications: first, the supernatant of each sample was diluted 1:10 with water prior to the reaction. For the glucose amount determination, the assay was scaled down to one third of the final volume recommended in the manual. The extinction was measured photometrically at λ = 340 nm using a Hitachi spectrometer U-2900. The acetate measurement was performed using the plate reader SpectraMax 340 (Molecular Devices) and 96 well plates (Greiner) loaded with a final volume of 200 μl of the assay mix. Glucose (Roth, Karlsruhe, Germany) added to cultures was sterilized by filter of 0.2 μm pore size.

### CFU determination

Overnight cultures of *S*. *aureus* SA113 were treated with 100-fold the MIC of DAP. Glucose was added at indicated time-points to final concentrations of 5 g/L (25 mM) unless stated otherwise. For the experiments with other compounds, glucose, fructose, ribose, xylose, glycerol, pyruvate, succinate, arginine or 2-deoxy-glucose were added at indicated time-points to final concentrations of 5 mM each. This concentration was chosen due to poorer aqueous solubility of some of the compounds compared to glucose. Viable counts were analyzed by CFU analysis on non-selective TSB agar plates. Cultures with viable counts below a threshold of 100 CFU/mL were judged as sterilized. All viable count experiments were conducted using at least three biological replicates.

### Statistical analysis

Data are expressed as mean ± SD. Statistical analysis is described for each experiment in the corresponding figure legend. For all comparisons, a P value of ≤0.05 indicated statistical significance. All statistical analyses were performed with GraphPad Prism version 4 and 5.

## Results

Cells of stationary growth phase *S*. *aureus* SA113 cultures maintain an active amino acid anabolism during high-level DAP treatment for several hours [23]. We assume that persister cells recalcitrant to eradication by this drug contribute significantly to this metabolic activity. Notably, a closer inspection of killing curves of our previous study suggested an increased killing efficiency by DAP after the addition of glucose. We here scrutinized this effect in more detail.

### Addition of glucose accelerates DAP-dependent killing of stationary growth phase *S*. *aureus in vitro*

**Fig 1:**
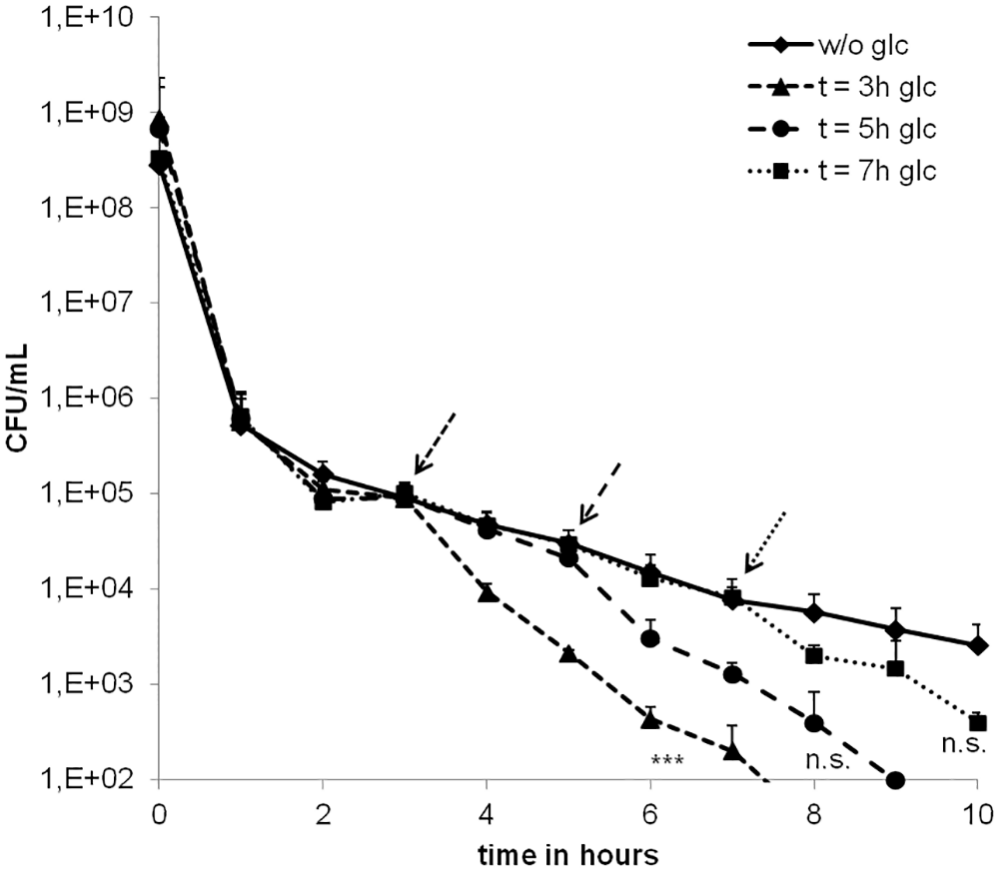
Time dependent killing of SA113. SA113 cells were treated with 100-fold the MIC of DAP starting at t = 0h. Glucose was added to cultures to final concentrations of 5 g/L each at time points 3h (triangles), 5h (circles), or 7h (squares), respectively, as indicated by arrows. CFU concentrations of a culture treated with DAP only (diamonds) were measured as a control. For statistical analysis area the delta of the CFU/mL was calculated 1 h and 4 h after glucose addition. Delta CFU/mL (n = 3) of glucose added after 3h (from 4h to 6h), 5h (6h to 8h) and 7h (8h to 10 h) and delta CFU/mL for the same time points of the control without Glc, were compared by 1-way ANOVA with Bonferroni's Multiple Comparison Test. ***p<0.001. n.s.: not significant.

Three SA113 cultures were grown identically in TSB medium to stationary growth phase at which the medium is depleted for glucose [23]. 100-fold the MIC of DAP was added at time point t = 0h to each of the cultures and at t = 3h, 5h or 7h, respectively, one culture each was supplemented with glucose at a final concentration of 5 g/L The viable counts indicated significantly enhanced killing when glucose was added at t = 3h compared to a culture challenged with DAP only (Fig 1). We now refer to the observation of enhanced DAP killing upon addition of glucose as the “Glc-DAP effect”. Between t = 0h and t = 1h, the number of CFU decreased to approximately 0.05% of the initial amount. Within the next two hours, the eradication slowed down, reflecting a typical biphasic killing behavior [9]. Of note, the addition of glucose three or five hours after DAP challenge resulted in an eradication of persister cells in the culture within the next five hours. In comparison, an SA113 culture exposed to 100-fold the MIC of DAP but no glucose still contained more than 1.6x10^3^ CFU/mL after ten hours. Cells residing in a DAP-tolerant state for a longer period (up to 7 hours after addition of the drug) also were susceptible to the Glc-DAP effect, albeit less pronounced. Notably, a comparable behavior was observed when stationary growth phase cells were washed and resuspended in PBS buffer, ruling out that other components in the spent medium are causative for the Glc-DAP effect (S1 Fig).

Upon supplementing the DAP-containing medium with lower concentrations of glucose (50 or 100 mg/L), killing of strain SA113 was only slightly affected, whereas higher glucose concentration markedly enhanced and killing efficiency (S2 Fig). The maximal effect was observed with 1 g/L glucose, whereas higher concentrations (up to 5 g/L) did not accelerate killing further. To account for glucose consumption we performed all further experiments using 5 g/L glucose.

### Glucose-enhanced killing of stationary growth phase *S*. *aureus* is DAP-specific and independent from cell division

At this point, it was conceivable that glucose induced killing of DAP challenged cells was the result of nutrient-dependent induction of cell division, rendering the cells generally more vulnerable to antibiotics. We have previously shown that stationary growth phase *S*. *aureus* cultures are extremely tolerant to a number of antibiotics *in vitro*, even at elevated drug-concentrations [35]. This was consistent with observations in the present study, in which drugs targeting the cell envelope (penicillin, 100-fold the MIC, 2 mg/L, or vancomycin, 100-fold the MIC, 400 mg/L) did not discernibly decrease the life count (S3 Fig). The addition of glucose did not induce killing by these antibiotics, which would be expected if the reversion to a replicating mode was responsible for the reinstated drug susceptibility. We therefore conclude that nutrient-dependent triggering of cell growth is not a reason for the Glc-DAP effect.

### Enhanced killing by DAP is specific to selected carbohydrates

**Fig 2:**
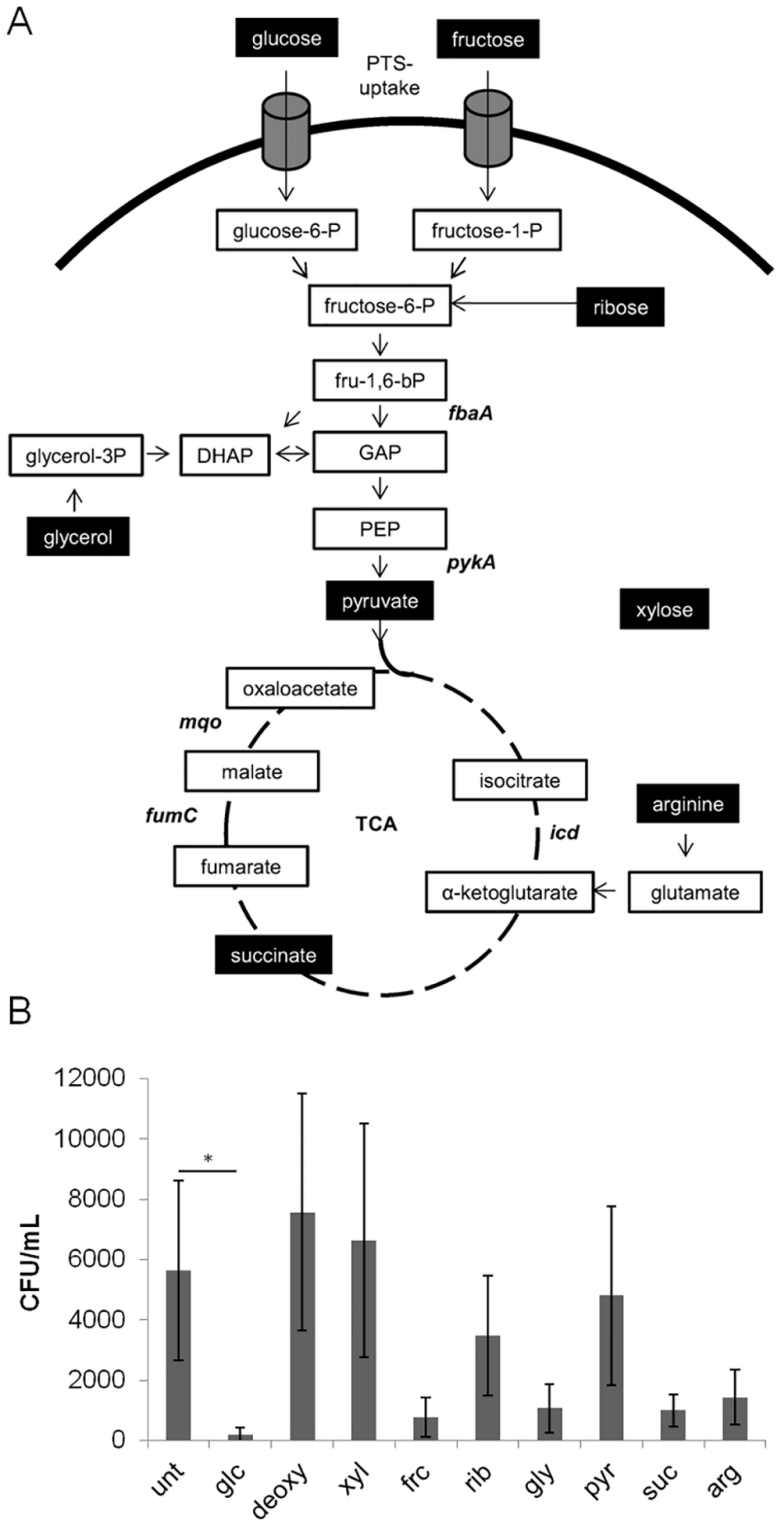
Influence of selected carbohydrates on DAP challenged *S*. *aureus* cultures. **A)** Schematic overview of tested metabolites and their entrance into the metabolism of *S*. *aureus* and genes responsible for selected metabolic reactions; fructose (fru), glyceraldehyde-3-phosphate (GAP), Dihydroxyacetone phosphate (DHAP), phosphoenolpyruvate (PEP). **B)** Selected compounds were added to final concentrations of 5 mM at t = 3h and the CFU values were determined after 24 hours. Groups were compared to the untreated control group by 1-way ANOVA with Dunnett's Multiple Comparison Test. *p<0.05.

Are other metabolites also capable of accelerating DAP-dependent killing of stationary growth phase *S*. *aureus*? We chose fructose, ribose, glycerol, pyruvate, succinate and arginine, all of which are substrates or intermediates of catabolic pathways or anaplerotic reactions of *S*. *aureus* (Fig 2A) to test this hypothesis. The compounds were added to final concentrations of 5 mM each three hours after the onset of DAP treatment. Xylose and 2-deoxy-glucose, which are both transported into the cytoplasm of *S*. *aureus* but are not further metabolized [52,53], served as controls. Killing was enhanced with fructose, glycerol, succinate and arginine, but only glucose showed a significant effect compared to the DAP-only control. Eradication kinetics were unaffected with xylose or 2-deoxy-glucose (Fig 2B).

As a further control, cultures were grown overnight in TSB-like medium in which glucose had been replaced by fructose. Also these were eradicated more efficiently by the addition of glucose, ruling out that an adaptation of metabolism to glucose during the cell cycle was mainly causative for the Glc-DAP effect (data not shown).

### The Glc-DAP effect is independent of the proton motive force

**Fig 3:**
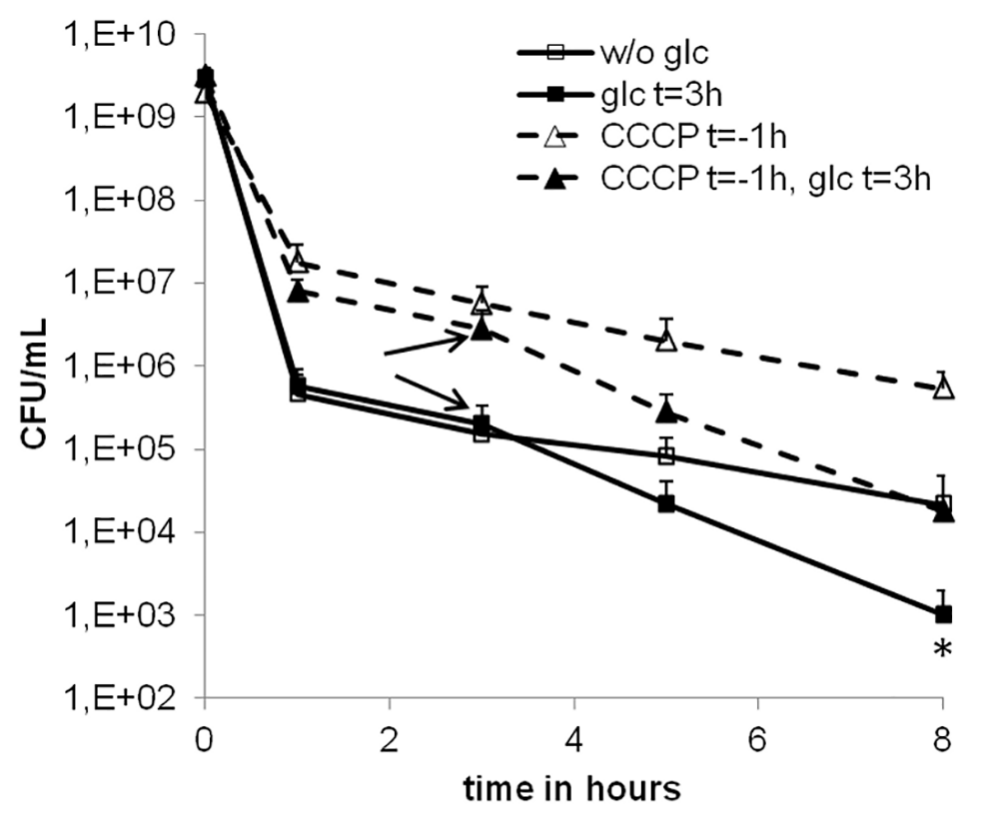
Influence of the proton motive force. CCCP pretreatment of DAP challenged SA113 cultures. Stationary phase SA113 cells were treated with 50 μM CCCP (triangles) at t = -1h, corresponding to one hour before the addition of 100-fold the MIC of DAP. At t = 3h (arrows), glucose was added to cultures (filled symbols). Cultures without CCCP pretreatment (squares) were handled identically. For statistical analysis endpoint ODs after eight hours were compared by 1-way ANOVA with Bonferroni's Multiple Comparison Test. *p<0.05.

The proton motive force (PMF) is generated by the electron transport chain and reduction equivalents originating from glycolysis and the TCA cycle. In order to examine a possible involvement of the PMF in the Glc-DAP effect, the uncoupler carbonyl cyanide m-chlorophenyl hydrazone (CCCP) was added to DAP challenged cells one hour before the addition of glucose. CCCP impedes energizing of membranes by scavenging protons, rapidly leading to a lack of ATP in the cell [54]. As shown previously, the activity of DAP against exponential growth phase *S*. *aureus* is unaffected by CCCP [32]. In our experiments, CCCP treatment of cultures during exponential growth phase ceased growth independent of DAP (S4 Fig). Treatment of cells with CCCP prior to the addition of DAP resulted in approximately 10-fold elevated persister levels compared to the untreated control (Fig 3), in agreement to results with CCCP-treated exponential phase *E*. *coli* cultures [55]. The Glc-DAP effect, however, was still observed in the presence of CCCP, suggesting a PMF independent mechanism.

### Analysis of *S*. *aureus* strains defective in glucose transport and catabolism

**Fig 4:**
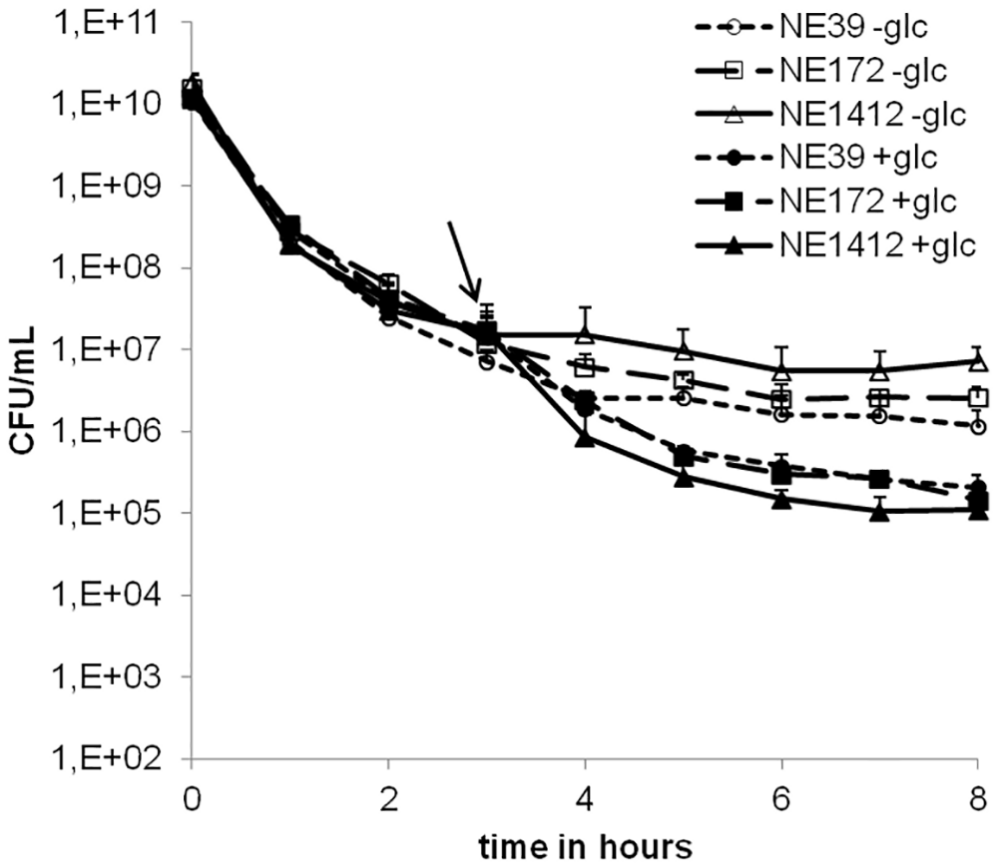
Time-dependent killing of PTS-mutants. Transposon mutants were treated with 250-fold the MIC of DAP. Glucose was added to the culture at t = 3h (arrow). NE39 (glucose-PTS specific IIABC component, circle), NE172 (glucose-PTS specific IIBC component domain protein, square). Strain NE1046 (formate dehydrogenase, triangle) served as control. For statistical analysis, the area under curve (AUC) was calculated from the time point of glucose (Glc) addition (3h). AUCs (n = 3) of all groups where compared by 1-way ANOVA with Bonferroni's Multiple Comparison Test, according to which the differences were not significant.

We next inspected selected steps of glucose transport and catabolism by exploiting specific mutant strains of the *S*. *aureus* USA300 JE2 based NARSA Transposon Mutant Library [47]. The first two chosen strains exhibited disrupted phosphotransferase (PTS) systems, namely the glucose-PTS specific IIABC component, SAUSA300_2476 (NE39) and the glucose-PTS specific IIBC component domain protein, SAUSA300_0191 (NE172). Compared to our PTS-positive control strain NE1046 (*fdhD*, formate dehydrogenase, SAUSA300_2231), both PTS mutants’ killing curves showed a less pronounced incongruity between challenge with Glc-DAP and DAP only (Fig 4). The reduced but still discernible Glc-DAP effect may be rationalized by redundant PTS systems or residual glucose transport due to secondary uptake systems of *S*. *aureus* [52]. The results hitherto suggest the involvement of one or more metabolic pathway(s) in the Glc-DAP phenomenon. In order to take a deeper look into central carbon metabolism, further transposon mutants affected at certain branch points of glycolysis, or TCA cycle, respectively, were examined (Fig 2A). Results obtained with these mutants were inconclusive as DAP susceptibility among the strains varied considerably even without glucose. However, a trend towards an accelerated killing of all the tested strains upon the addition of glucose was observed (S5 Fig).

### The Glc-DAP effect is not affected by physiologic changes in the pH

The interplay between oxygen supply and an excess of glucose can lead to an overload of metabolic pathways [56]. In *S*. *aureus* this results in accumulation of acetate and lactate stemming from pyruvate and concomitant acidification of the medium [57]. We determined both the pH value and the amount of acetate in DAP-containing cultures before and after the addition of glucose. The medium became slightly alkalized during the first three hours of DAP treatment (S6A Fig). After the addition of glucose the pH value rapidly decreased from 7.8 to about 7.4 and then leveled off, arguably due to glucose metabolism. The concentration of acetate was stable for the first three hours and rose upon glucose addition (S6B Fig). An artificial adjustment of the pH value in the medium, to resemble the glucose-dependent changes, did not influence the killing behavior by DAP only (data not shown). Thus, it is unlikely that acidification may be causative of the Glc-DAP effect.

### Strains less susceptible to DAP are also subject to the Glc-DAP effect

**Fig 5:**
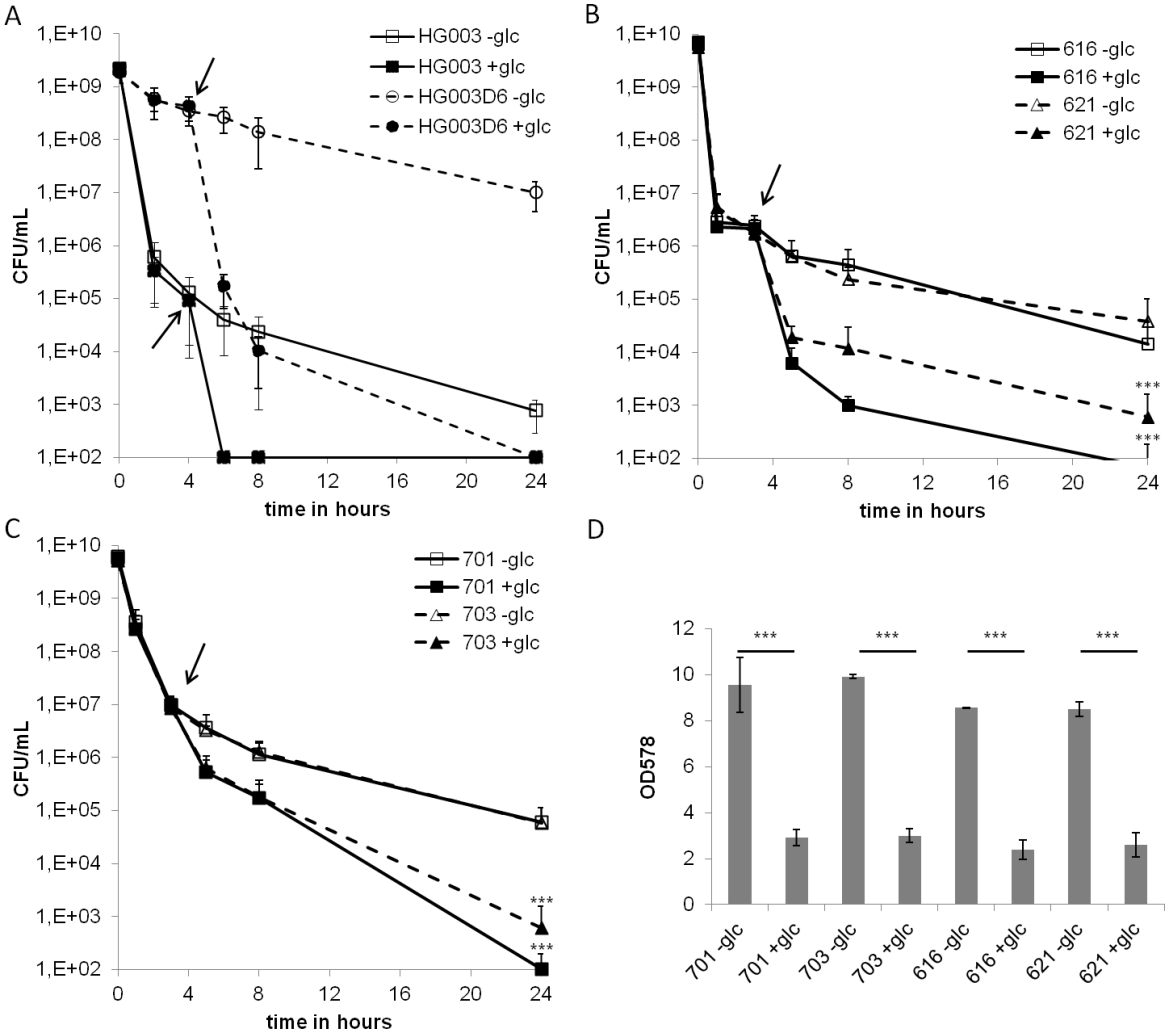
Time-dependent killing of strains with different susceptibilities towards DAP. Stationary phase cultures of HG003, HG003 D6 and the clinical *S*. *aureus* strains 616, 621, 701 and 703 were treated with 100-fold, or 250-fold the MIC of DAP, respectively. At t = 3h, glucose was added (arrow) to the cultures (filled symbols) and CFU values were determined over time. For statistical analysis endpoint ODs after 24 hours for -Glc and +Glc for each strain were compared with unpaired t-test (with Welch’s correction for unequal variances if appropriate) ***p<0.001. **A)** Growth-phase-dependent DAP-tolerant strain HG003 D6 (square) and parent wild type strain HG003 (triangle). **B)** DAP-tolerant strains 701 (square) and 703 (triangle) (MIC = 2 mg/L). **C)** Sensitive strains 616 (square) and 621 (triangle) (MIC = 0.5 mg/L). **D**) Optical densities after 24h of incubation. Strains were challenged with 100-fold the MIC of DAP from t = 0h. Identically treated cultures were supplemented with glucose from t = 3h.

We next investigated, whether the addition of glucose also increases DAP-dependent killing of strains with decreased susceptibility to this drug. Strain HG003 D6 [48] had previously been isolated as a highly DAP-tolerant mutant generated by cyclic treatment with high doses of the drug. The addition of glucose and DAP led to a tremendous killing also of this strain (Fig 5A) whereas the culture treated with DAP only still contained more than 1x 10^8^ CFU/mL seven hours after drug-addition. The parent strain HG003 was highly susceptible for the Glc-DAP effect reflecting a more pronounced killing behavior than SA113 (Fig 1). In addition, two DAP sensitive strains (616, 621; MIC = 0.5 mg/L) and two less DAP-susceptible strains (701, 703; MIC = 2 mg/L), all isolated from a patient with relapsing endocarditis during DAP therapy [42,58] were tested for the Glc-DAP effect. Intriguingly, the killing efficiency was increased about 100-fold (strain 703) and 600-fold (strain 701) compared to the treatment without glucose after 24 hours of incubation (Fig 5B). Similar results were obtained for the two less tolerant strains 616 and 621 (Fig 5C). While DAP is regarded as non-lytic against *S*. *aureus* [59], the experiments with strains 616, 621, 701 and 703 revealed a drastic decrease in OD_578_ values after a 24 hour period of Glc-DAP treatment (Fig 5D). The OD_578_ of the samples treated with Glc-DAP decreased by more than 75% compared to the cultures treated with DAP only. This apparent cell lysis was observed with all four strains, irrespective of their sensitivity towards the drug.

## Discussion

Metabolite induced killing of bacterial persisters has been associated with PMF generation and concomitant uptake of aminoglycoside compounds [20,45,46]. We here show that the lipopeptide antibiotic DAP also exhibits enhanced killing efficiency of *S*. *aureus* in the presence of glucose. Of note, this effect was also observed with a number of strains with low DAP-susceptibility. Our experiments with the uncoupler CCCP furthermore indicate that the Glc-DAP effect is not merely a consequence of PMF generation. Instead, the metabolism of glucose appears to be crucial for the Glc-DAP effect, which was neither prominent with low concentrations of glucose, nor with the non-metabolizable 2-deoxy-glucose. The observations of our study are consistent with two hypotheses for the mechanistic basis of the Glc-DAP effect. The first one suggests an influence of Glc-transport proteins on DAP’s mode of action, while the second is based upon Glc induced and DAP-specific lysis of cells. Based upon our observations with a number of *S*. *aureus* transposon-mutants, it is conceivable that components of the Glc PTS transport system may serve as receptors or targets of DAP. Accordingly, Glc-dependent induction of specific PTS transporters [60,61] will increase susceptibility to DAP. A similar effect was observed in *Lactococcus lactis* with bacteriocins that share biochemical features with DAP [62]. Moreover, mutations in PTS proteins confer a high degree of DAP-non-susceptibility in *Enterococcus faecium* [63]. Regarding the integrity of *S*. *aureus* cells upon DAP treatment, contradictory results have been reported. Electron microscopic images have shown tremendous morphological changes of *S*. *aureus* cells during DAP challenge, but not lysis [64], in agreement with another study also suggesting a lysis-independent mechanism of this drug [59]. However, autolysis after DAP addition was observed, at least partially, in some *S*. *aureus* strains during exponential phase [65]. Cell lysis may be augmented by intrinsic murein hydrolases. A potentiated lysis of exponential phase *Staphylococcus cohnii* upon addition of glucose was described with Pep 5, a cationic bactericidal peptide produced by *Staphylococcus*. *epidermidis* 5 [66]. It is conceivable that carbohydrates in combination with specific drugs induce a suicidal mechanism in persister cells comparable to programmed cell death [67]. Of note, carbohydrate metabolism influences murein hydrolase activity in *S*. *aureus* [68,69]. For example, the pleiotropic regulator CcpA activates transcription of the hydrolase activator *cidA* in the presence of glucose [70]. *cidA* is part of the *cidABC* operon which together with *lrgAB* is involved in the regulation of murein hydrolase activity and autolysis [69,71,72]. According to our previous data, an upregulation of the TCA cycle activity may lead to an overflow metabolism [23] and acetate derived from pyruvate activates *cidABC* and *lrgAB* transcription. Further studies are required to verify these speculations in regard to the Glc-DAP effect.

Recently, a resuscitation promoting factor of *S*. *aureus* was postulated that is involved in shifting dormant cells back to a dividing state [73]. This factor can be ruled out as responsible for the Glc-DAP effect which we also observed in buffered solution, devoid of components found in culture supernatants. Although the regulatory network of hydrolase activity is still not well understood, it should be considered as a potential target for the development of new anti-persister therapies of *S*. *aureus*. Artificial activation of peptidoglycan hydrolases could thereby lead to a random lysis process with fatal consequences for the cells independent of both the susceptibility towards antibiotics and their physiological state. It would be interesting to investigate the significance of the Glc-DAP effect in the treatment of staphylococcal infection. Notably, our experiments were based upon *in vitro* stationary growth phase cultures that were challenged with DAP concentrations that exceed serum concentrations in patients treated with this drug by more than tenfold [74,75]. Certainly, the systemic application of glucose to enhance DAP-dependent killing of *S*. *aureus* persisters in a patient is limited, as the blood sugar level in the human body is normally subject to homeostatic regulation. Of note, glycemia of non-diabetic humans is in a comparable range as the glucose concentrations that we determined to enhance DAP’s function. The importance of glucose as an adjuvant for DAP may thus have gone unnoticed in patients so far. It may be an option to improve DAP-efficiency in the treatment of non-invasive acute bacterial skin and skin-structure infections by increasing local concentrations of glucose. Hopefully, recent achievements regarding the eradication of persister cells will also aid in reducing the formation of drug resistant cells that pose an ever-growing issue in public and clinical health [4,39,76–78].

## Supporting Information

S1 Fig Time-dependent killing of SA113 cells in PBS. Stationary phase SA113 cells grown in TSB were harvested and resuspended in PBS. At t = 0h, 100-fold the MIC of DAP was added to the cell suspensions. At t = 3h, one cell suspension was supplemented with glucose (filled square), the other was left unaffected (open square) and CFU concentrations were determined over time.
(TIF)

S2 Fig Influence of the glucose concentration on the efficiency of the Glc-DAP effect. Stationary phase SA113 cells were treated with 100-fold the MIC of DAP at t = 0h. At t = 3h, different amounts of glucose were added and CFU values were determined after another four hours. Pearson’s r coefficient: r = -0,704.
(TIF)

S3 Fig Penicillin and vancomycin treatment of SA113 ± glucose. Stationary phase SA113 cells were treated with 100-fold the MIC of penicillin (square) or 100-fold the MIC of vancomycin (triangle) at t = 0h. Glucose was added at t = 3h (arrow and filled symbols).
(TIF)

S4 Fig Effect of CCCP on growth of SA113. SA113 was grown in TSB supplemented with glucose (t = 0h, squares), 100 μM CCCP (t = 0h, diamonds), glucose and CCCP (t = 0h, triangles), or glucose (t = 0h) and CCCP (t = 3h) (circles), respectively.
(TIF)

S5 Fig Investigation of enzymatic branch points in glycolysis and TCA cycle. Time dependent killing of stationary phase cultures with 250-fold the MIC of DAP. NE427 (*fumC*^*-*^, fumarate hydratase, diamonds), NE476 (*fba*^*-*^, fructose bisphosphate aldolase, squares), NE491 (*icd-*, isocitrate dehydrogenase, triangles), NE1003 (*mqo*^*-*^, malate-quinone oxidoreductase, x-mark), NE1046 (*fdh*^*-*^, formate dehydrogenase, circles), NE1407 (*pyk*^*-*^, pyruvate kinase, asterisks). For statistical analysis area under curve (AUC) was calculated from the time point of glucose (Glc) addition (3h). AUCs (n = 3) of all groups where compared to NE1046 *fdh* by 1-way ANOVA with Dunnett's Multiple Comparison Test.
(TIF)

S6 Fig pH and acetate/glucose measurement. Cultures were treated with 100-fold the MIC of DAP at t = 0h. **A)** Glucose was added (filled squares) at t = 3h (arrow) and pH values were determined over time. **B)** Acetate (triangle) and glucose (square) measurement of a culture with glucose added at t = 3h.
(TIF)
