# Supplementary figures and images for: Glucose Augments Killing Efficiency of Daptomycin Challenged Staphylococcus aureus Persisters
Source: PLoS One. 2016 Mar 9;11(3):e0150907. doi: 10.1371/journal.pone.0150907 (PMC4784881; doi:10.1371/journal.pone.0150907)

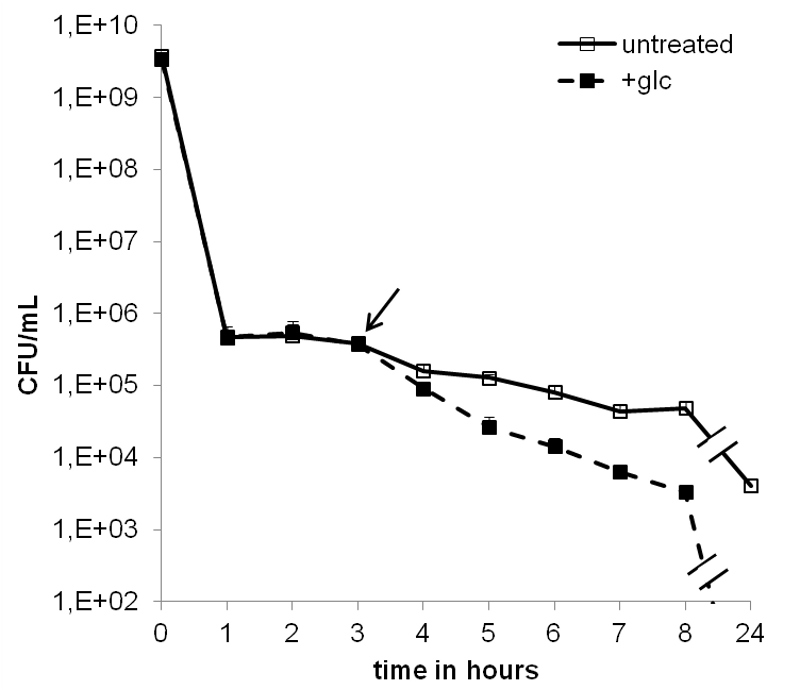

Supplement: S1 Fig — Stationary phase SA113 cells grown in TSB were harvested and resuspended in PBS. At t = 0h, 100-fold the MIC of DAP was added to the cell suspensions. At t = 3h, one cell suspension was supplemented with glucose (filled square), the other was left unaffected (open square) and CFU concentrations were determined over time. (TIF) [file pone.0150907.s001.tif]

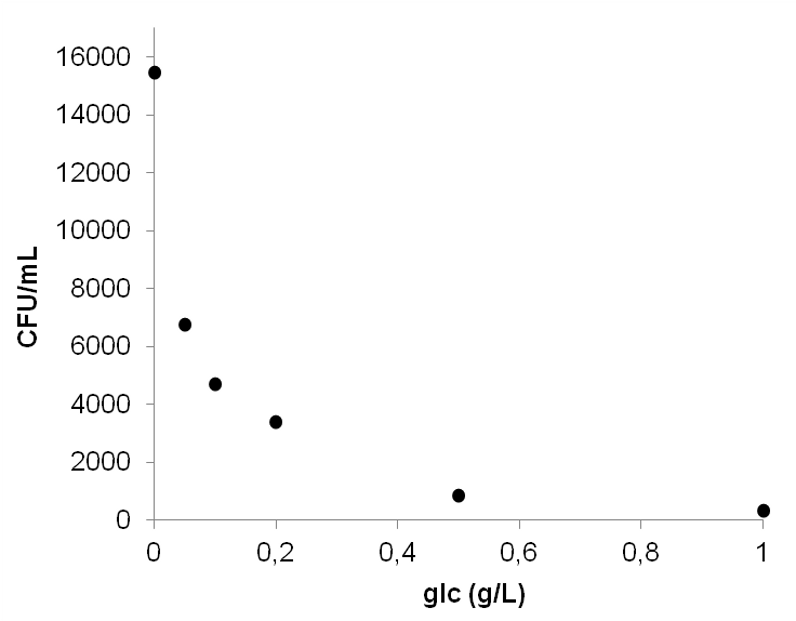

Supplement: S2 Fig — Stationary phase SA113 cells were treated with 100-fold the MIC of DAP at t = 0h. At t = 3h, different amounts of glucose were added and CFU values were determined after another four hours. Pearson’s r coefficient: r = -0,704. (TIF) [file pone.0150907.s002.tif]

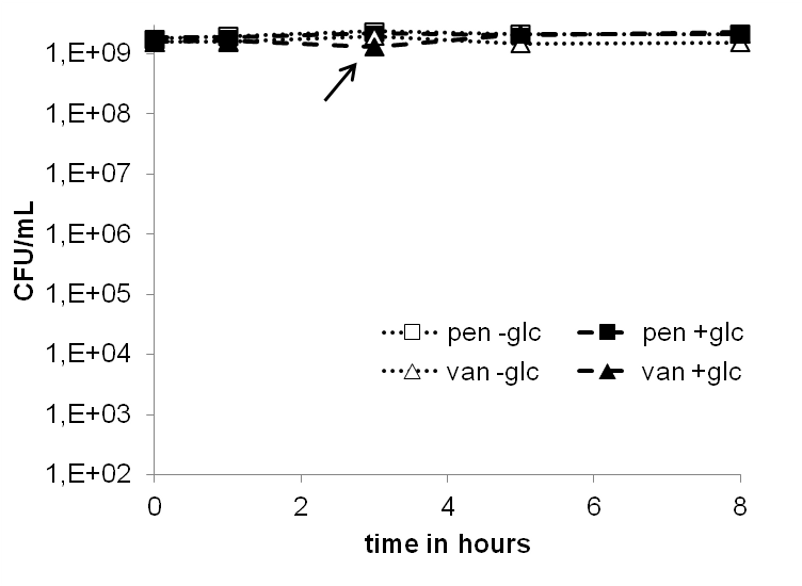

Supplement: S3 Fig — Stationary phase SA113 cells were treated with 100-fold the MIC of penicillin (square) or 100-fold the MIC of vancomycin (triangle) at t = 0h. Glucose was added at t = 3h (arrow and filled symbols). (TIF) [file pone.0150907.s003.tif]

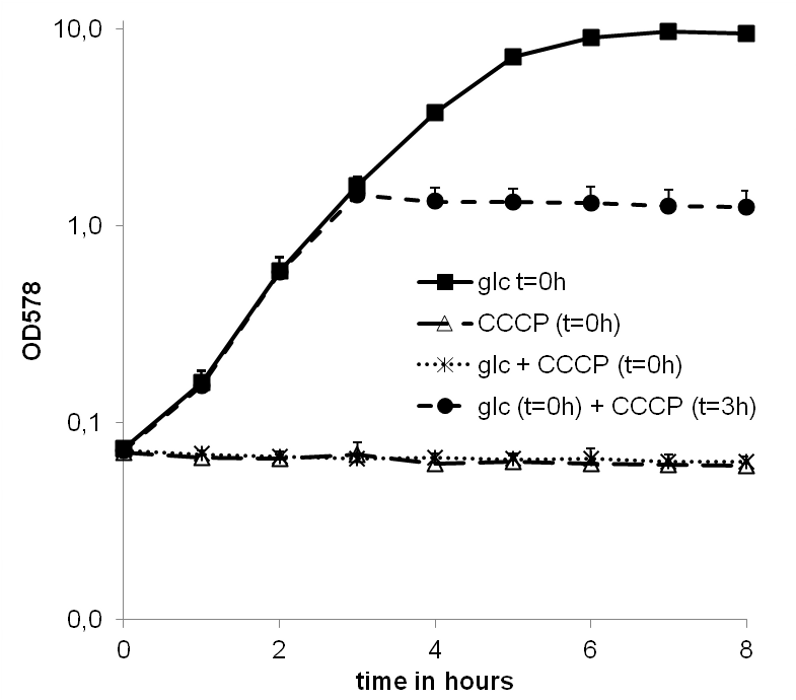

Supplement: S4 Fig — SA113 was grown in TSB supplemented with glucose (t = 0h, squares), 100 μM CCCP (t = 0h, diamonds), glucose and CCCP (t = 0h, triangles), or glucose (t = 0h) and CCCP (t = 3h) (circles), respectively. (TIF) [file pone.0150907.s004.tif]

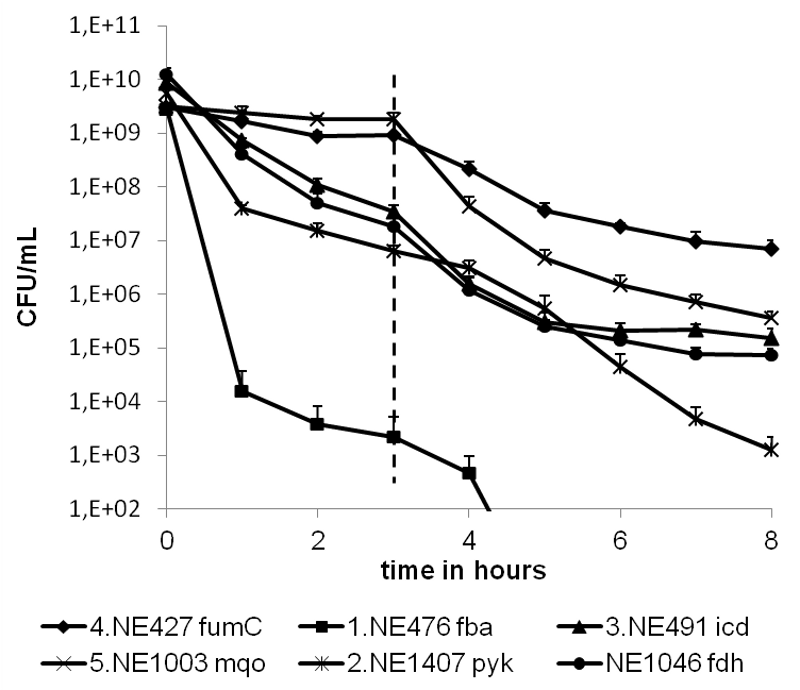

Supplement: S5 Fig — Time dependent killing of stationary phase cultures with 250-fold the MIC of DAP. NE427 (fumC-, fumarate hydratase, diamonds), NE476 (fba-, fructose bisphosphate aldolase, squares), NE491 (icd-, isocitrate dehydrogenase, triangles), NE1003 (mqo-, malate-quinone oxidoreductase, x-mark), NE1046 (fdh-, formate dehydrogenase, circles), NE1407 (pyk-, pyruvate kinase, asterisks). For statistical analysis area under curve (AUC) was calculated from the time point of glucose (Glc) addition (3h). AUCs (n = 3) of all groups where compared to NE1046 fdh by 1-way ANOVA with Dunnett's Multiple Comparison Test. (TIF) [file pone.0150907.s005.tif]

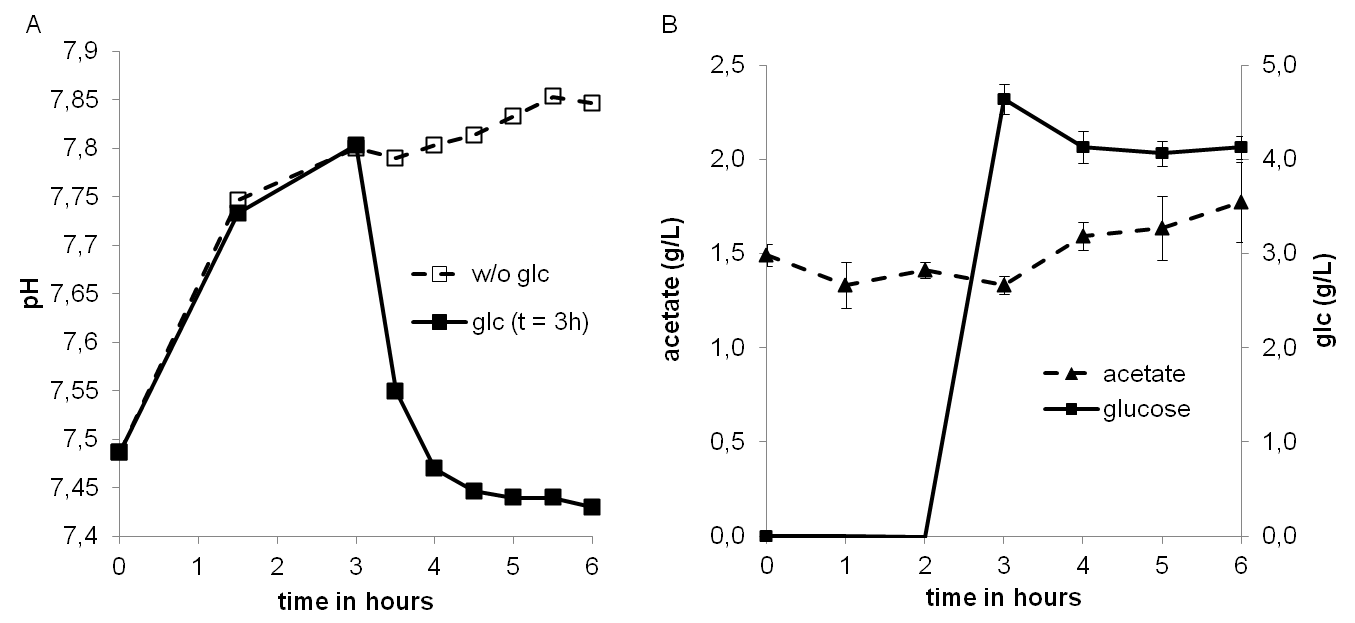

Supplement: S6 Fig — Cultures were treated with 100-fold the MIC of DAP at t = 0h. A) Glucose was added (filled squares) at t = 3h (arrow) and pH values were determined over time. B) Acetate (triangle) and glucose (square) measurement of a culture with glucose added at t = 3h. (TIF) [file pone.0150907.s006.tif]
